# Associations between readmission and patient-reported measures in acute psychiatric inpatients: a study protocol for a multicenter prospective longitudinal study (the ePOP-J study)

**DOI:** 10.1186/s13033-019-0298-3

**Published:** 2019-06-07

**Authors:** Sosei Yamaguchi, Yasutaka Ojio, Junko Koike, Asami Matsunaga, Makoto Ogawa, Hisateru Tachimori, Akiko Kikuchi, Hiroshi Kimura, Ataru Inagaki, Hiroyuki Watanabe, Yoshiki Kishi, Koji Yoshida, Takaaki Hirooka, Satoru Oishi, Yasuhiro Matsuda, Chiyo Fujii

**Affiliations:** 1 Department of Community Mental Health & Law, National Institute of Mental Health, National Center of Neurology and Psychiatry, 4-1-1 Ogawa-Higashi, Kodaira, 187-8553 Japan; 2 Translational Medical Center, National Center of Neurology and Psychiatry, 4-1-1 Ogawa-Higashi, Kodaira, 187-8553 Japan; 3 The Institute for Global Health Policy Research, Bureau of International Health Cooperation, National Center for Global Health and Medicine, 1-21-1 Toyama Shinjuku-ku, Tokyo, 162-8655 Japan; 4 Department of Psychiatry, Graduate School of Medicine, Chiba University, 1-8-1 Inohana, Chuo-ku, Chiba, 260-8670 Japan; 5 Department of Psychiatry, Gakuji-kai, Kimura Hospital, 6-19 Higashi-honcho, Chuo-ku, Chiba, 260-8670 Japan; 6 College of Education, Psychology and Human Studies, Aoyama Gakuin University, 4-4-25 Shibuya, Shibuya-ku, Tokyo, 150-8366 Japan; 7 Division of Medical Treatment and Rehabilitation, Center of Forensic Mental Health, Chiba University, 1-8-1 Inohana, Chuo-ku, Chiba, 260-8670 Japan; 8 Department of Psychiatry, Okayama Psychiatric Medical Center, 3-16 Shikata-Honmachi, Kita-ku, Okayama, Japan; 9 Department of Human Care and Support, Toyo University, 48-1 Oka, Asaka, 351-8510 Japan; 10 Department of Psychiatry, Kitasato University School of Medicine, 1-15-1 Kitazato, Minami, Sagamihara, 252-0374 Japan; 11 Department of Psychiatry, Nara Medical University School of Medicine, 840 Shijo, Kashihara, 634-8521 Japan

**Keywords:** Longitudinal study, Patient-reported experience, Patient-reported outcome, Psychiatric hospital, Readmission

## Abstract

**Background:**

Several previous observational studies have reported the risk factors associated with readmission in people with mental illness. While patient-reported experiences and outcomes have become increasingly important in healthcare, only a few studies have examined these parameters in terms of their direct association with readmission in an acute psychiatric setting. This project will investigate multiple factors associated with readmission and community living in acute psychiatric patients in Japan. This study will primarily investigate whether patient-reported experiences at discharge, particularly quality of life (QoL), are associated with future readmission and whether readmission after the index hospitalization is associated with changes in patient-reported outcomes during the study period. Here, we describe the rationale and methods of this study.

**Methods:**

This multicenter prospective cohort study is being conducted in 21 participating Japanese hospitals, with a target sample of approximately 600 participants admitted to the acute psychiatric ward. The study has four planned assessment points: time of index admission (T1), time of discharge (from the index admission) (T2), 6 months after discharge from the index admission (T3), and 12 months after discharge from the index admission (T4). Participants will complete self-reported measures including a QoL scale, a subjective disability scale, and an empowerment- and self-agency-related scale at each assessment point; additionally, service satisfaction, subjective view of need for services, and subjective relationships with family members will be assessed at T2 and T3. We will assess the participants’ hospitalization during the study period and evaluate several potential individual- and service-level factors associated with readmission and patient-reported experiences and outcomes. Multivariate analyses will be conducted to identify potential associations between readmission and patient-reported experiences and outcomes.

**Discussion:**

The present study may produce evidence on how patient-reported experiences at discharge influence readmission and on the influence of readmission on the course of patient-reported outcomes from admission to community living after discharge. The study may contribute to improving care for both patients’ subjective views of their own health conditions and their community lives in an acute psychiatric setting.

*Trial registration* University Hospital Medical Information Network—Clinical Trials Registry (UMIN-CTR) UMIN000034220. Registered on September 20, 2018.

**Electronic supplementary material:**

The online version of this article (10.1186/s13033-019-0298-3) contains supplementary material, which is available to authorized users.

## Background

Over the past 50 years, there have been two predominant themes in psychiatric care worldwide, namely, community living and personal recovery for people with mental illness, which focus on the individual processes through which people with mental illness subjectively achieve meaningful lives [1, 2]. With regard to community living, as the number of patients who live in the community has increased through deinstitutionalization in developed countries [3–5], several studies have examined factors associated with readmission, which has a negative impact on sustainable community living for people with mental illness [6–16]. Previous relevant studies and systematic reviews have confirmed several individual- and service-level factors associated with readmission [6–16]. Individual-level factors include diagnosis, sex, age, treatment adherence, symptoms, housing problems, problematic behaviors, past hospitalization, and comorbidities. Service-level factors include inpatient care programs, medications, provision of care after discharge, other types of comprehensive community care and locations of hospitals and patients [11–16]. In addition, a recent study that sought to identify the various risks for readmission developed and validated a comprehensive index called the READMIT, whose score was associated with future readmission among psychiatric inpatients [17].

While several objective factors at patient discharge have been reported to be risks for future readmission, patients’ subjective experiences may also be associated with readmission. If patients’ subjective views of themselves at discharge or their experiences during hospitalization can be shown to be independent risk factors for their future readmission, psychiatric care should focus on improving these factors. Indeed, patient-reported assessments of QoL and personal recovery have been somewhat correlated with symptoms and functions [18, 19]. In addition, one recent study reported a potential association between subjective QoL and future readmission among patients with schizophrenia in Israel [20]. However, a systematic review also noted that few studies have examined the association between subjective patient views and readmission [11]. Therefore, the relationship between these variables is still hypothetical.

As another example of subjective experience and readmission, psychiatric patients may experience traumatic treatment during their hospitalization, particularly in an acute care or compulsory treatment settings [21, 22]. Poor service engagement caused by negative perceptions of the treatment received is likely to adversely affect clinical outcomes [23]. Indeed, in a large-scale longitudinal study, satisfaction with inpatient care was associated with readmission in acute inpatients with schizophrenia [24]. However, a systematic review reported inconsistent findings regarding the associations between patients’ views of care quality and prognostic outcomes, including readmission [22]. In short, there is still limited evidence on the relationship between patient-reported treatment experiences and readmission.

In parallel with the personal recovery movement worldwide, the interest in patient-reported outcomes has dramatically increased in psychiatric care [19, 25–28]. Conceptually, the personal recovery process does not end even when a patient faces a crisis linked to readmission [29]. However, a small number of studies have examined whether readmission affects changes in patient-reported outcomes or reported the potential factors that affect patient-reported outcomes after hospital discharge. For example, one German study did not find any variables that directly affected changes in QoL scores between admission and 9-month follow-up in schizophrenia inpatients, but the study suggested that depressive symptoms were a potential factor [30]. Another German randomized control trial of a psychoeducation and monitoring program reported the potential influence of compulsory rehospitalization on changes in self-reported mental health functioning during a 2-year follow-up [31]. In addition, past studies in inpatient and outpatient settings identified factors potentially associated with changes in QoL or empowerment, including age, male sex, diagnosis (e.g., schizophrenia and bipolar disorders), and length of hospital stay [31–33], although patient-reported outcomes, particularly QoL, are likely to vary among post discharge residential service settings, such as outreach services [34, 35]. In sum, while past studies have identified potential factors that influence the prognostic value of patient-reported outcomes, there is still insufficient evidence on the direct association between changes in patient-reported outcomes (e.g., QoL, empowerment, and self-reported functional impairment) and readmission, particularly among patients in the acute phase.

In terms of the Japanese context, Japan is undergoing a transition from inpatient care to community mental health care. Most psychiatric care is still provided in inpatient settings [36]; the average length of psychiatric hospitalization is approximately 300 days [37]. The Japanese government has reformed relevant laws to prevent long-term inpatient care starting in the 21st century. Clinicians have started to develop systems for community care [37, 38]. These changes have resulted in the fact that more than 80% of inpatients were discharged within 1 year [39]. Nevertheless, approximately 40% of inpatients are rehospitalized within 1 year after discharge [38]. Some recent studies in Japan based on analyses of the national database have noted that highly intensive medical support delivered as part of inpatient care and follow-up visits has potentially reduced the risk of readmission [40, 41]. However, most Japanese empirical studies on psychiatric readmission have focused on the system level. Few studies have comprehensively examined either the individual factors that predict readmission after discharge or patient-reported outcomes after discharge, particularly in acute psychiatric care settings. In other words, both clinical variables and patient-reported measures related to hospitalization and community living in Japan remain unclear.

## Aims and objectives

To address the evidence gap, we initiated the ePOP-J study (early discharge and Prognostic community Outcomes for Psychiatric inpatients in Japan: a longitudinal study), which is a multicenter prospective cohort study designed to investigate multiple factors, including patient-reported measures, associated with readmission and community living, in acute psychiatric patients in Japan. The overarching aim of the present study is to explore the association between patient-reported experiences and outcomes and the incidence of readmission during 12 months of follow-up. In this study, patient-reported measures, particularly QoL, will be used to assess both exposures and outcomes. The study primarily aims to evaluate the following associations and factors, although we will also perform a comprehensive exploratory analysis to identify other (non-patient-reported) factors for readmission after evaluating the described associations and factors:

1. The relationship between readmission during the 12-month follow-up beginning at discharge after the index admission (primary outcome), and the QoL score at discharge (primary exposure).
2. The association between readmission during the 12-month follow-up beginning at discharge after the index admission and the self-reported disability score, empowerment/self-agency-related measure score or inpatient care service satisfaction score at discharge.
3. Influence of readmission on changes in scores for patient-reported outcome measures (QoL, self-reported disability and empowerment/self-agency-related outcome) during the study period.
4. Other potential factors influencing score changes in patient-reported outcome measures, such as age, sex, diagnosis, length of hospital stay, living conditions, and utilization of outreach services after discharge.

## Methods and analysis

### Study design and settings

**Fig. 1:**
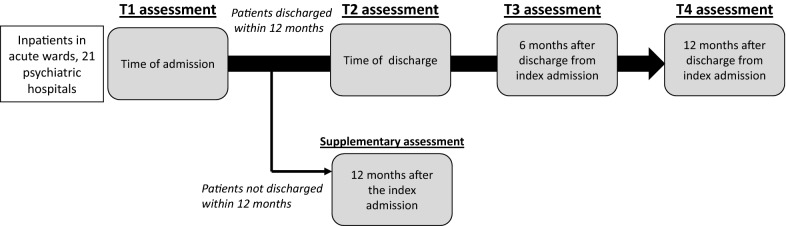
Overall design and process of the study

A multicenter prospective cohort study has been planned. The study protocol was designed by professional researchers, clinicians, and mental health services users. Specifically, our project team has involved a mental health service user researcher as a core member, and we collaboratively determined the study participants’ eligibility criteria, recruitment methods, and outcomes and exposures. We employed a prospective design for this study to repeatedly assesses patient-reported experiences and outcomes in situations where these measures are not routinely evaluated in the current mental health care system in Japan. We recruited 21 psychiatric hospitals with acute wards. Because human resources that perform research work vary considerably among psychiatric hospitals in Japan, we needed to recruit only hospitals with sufficient staff members to conduct a prospective study. Therefore, we used a snowball sampling method across Japan instead of using random sampling. The study will assess acute ward inpatients’ exposures and outcomes at admission (T1), discharge (T2), 6 months after discharge from the index admission (T3), and 12 months after discharge from the index admission (T4). We will also evaluate exposures and outcomes among inpatients who cannot be discharged within 12 months of the index admission (Fig. 1). Patient recruitment began on 1 October 2018.

### Eligibility criteria

#### Inclusion criteria

Eligible participants are patients diagnosed with psychiatric disorders based on the International Classification of Diseases 10th revision (F00–F90) who require treatment in the acute psychiatric ward of participating psychiatric hospitals between 1 October 2018 and 30 September 2019. The main analysis will include only participants discharged from each participating hospital within 12 months.

#### Exclusion criteria

Exclusion criteria are as follows: age younger than 20 years or older than 60 years and hospitalization for specific tests and treatments (e.g., psychiatric testimony, electroconvulsive therapy). Patients are also excluded if they are immediately moved to other medical departments for long-term care after hospitalization (e.g., if a severe physical disorder was found) or if they receive psychiatric treatment outside of participating hospitals or their affiliated medical institutions.

### Procedures for informed consent

When a potential participant is admitted to the acute ward of a participating psychiatric hospital, staff members, including psychiatrists, nurses, occupational therapists, and social workers, screen the patient against the eligibility criteria. If an inpatient fulfils all the criteria and has the cognitive capacity for informed consent, trained staff members will provide a description of the study and the ethical issues involved using the descriptive brochure approved by the ethics committee. Informed consent will be obtained from the participant as soon as possible, within approximately 2 weeks after admission. Each hospital will recruit a maximum of 10 participants per month.

### Procedures for data collection

After informed consent is obtained, participants will complete a self-administered questionnaire at the time of admission (T1). The staff members will assist by reading the questionnaire if necessary. Trained staff members will also complete the first assessment sheet, which includes sociodemographic information and past medical history, based on medical records. In addition, they will assess staff-rated measures based on observation and a brief interview with the participant. At discharge (T2), the participant will complete the self-administered questionnaire again. Next, staff members will complete staff-rated measures and will document the types of inpatient care that were provided during hospitalization on the second assessment sheet, based on medical records. At 6 months (T3) and 12 months (T4) after the index admission, the participant will complete a self-administered questionnaire, and staff members will complete an assessment sheet detailing staff-rated measures, readmission, and outpatient services. To prevent loss to follow-up at 6 months (T3) and 12 months (T4), staff members in the inpatient care department, outpatient care department, and outreach team will cooperate to share information on each participant’s status.

For participants who are not discharged within 12 months of the index admission, the same procedure used for the discharge survey (T2) will be conducted when the hospital stay reaches 365 days. At this time, the patient’s survey will be concluded. The main analysis will only include participants who are discharged within 12 months.

Data monitoring and management will be conducted by the Department of Community Mental Health and Law, National Center of Neurology and Psychiatry, which is independent from the grant funder. At least two researchers or research assistants will check the data to maintain data quality.

### Research measures and valuables

The variables assessed at each time point in this study are summarized in Table 1.

**Table 1:** Collection of data on exposures, outcomes, and other explanatory variables at each assessment point

Item	Time of admission (T1)	Time of discharge (T2)	6-month follow-up (T3)	12-month follow-up (T4)
Patient-reported outcomes/experiences				
Quality of life (EQ-5D-5L)	✓	✓	✓	✓
Perception of disabilities (SDS-J)	✓	✓	✓	✓
Empowerment and self-agency related scale	✓	✓	✓	✓
Satisfaction with care		✓	✓	
Subjective relationship with family		✓	✓	
Clinical and social variables				
Past hospitalisation or readmission				
Number of admissions^a^	✓	✓	✓	✓
Length of hospitalisation^a^	✓	✓	✓	✓
Type of hospitalisation (voluntary/involuntary)^a^	✓	✓	✓	✓
Reasons for emergency admission^a^	✓	✓	✓	✓
Unplanned discharge from index admission^a^		✓		
Psychiatric present status^b,c^	✓			
Symptoms (1 item)	✓	✓	✓	✓
Social relationships (1 item)	✓		✓	✓
Employment status	✓	✓	✓	✓
Functioning (PSP)	✓	✓	✓	✓
History of problematic behaviours^b^	✓	✓	✓	✓
Risk of problematic behaviours	✓	✓	✓	✓
Weight	✓	✓	✓	✓
Body mass index	✓	✓	✓	✓
Other exposure variables				
Socio-demographics				
Age^a^, sex, living condition, living location, social security etc.	✓			
Diagnostic information^a^				
Psychiatric disorders^a^	✓	✓		
Physical disorders^a^	✓	✓		
Past utilisation of services				
Emergency services^b^	✓			
Police or administrative interventions	✓			
Community medical services^b^	✓		✓	✓
Outpatient services^a,b^	✓		✓	✓
Social and welfare services^b^	✓		✓	✓
Case management or outreach services	✓	✓	✓	✓
Crisis planning^b^	✓	✓	✓	✓
Prescriptions	✓	✓	✓	✓
Inpatient care during hospitalisation				
Seclusion order		✓		
Restraint order		✓		
Electroconvulsive therapy^b^		✓		
Discharge plan or planning^a,b^		✓		
Therapies specific to hospitalisation^b,d^		✓		
Outpatient care during follow-up				
Outpatient service visit within 7 days of discharge from index admission			✓	
Regular outpatient service visits			✓	✓
Online medical examinations			✓	✓
Local social and welfare resources				
Number of social and welfare service agencies in the patient’s area of residence				✓

*EQ-5D-5L* EuroQol five dimensions and five levels, *PSP* Personal and Social Performance scale, *SDS-J* Sheehan Disability Scale—Japanese version

^a^Component of the READMIT index

^b^Possible responses are YES/NO

^c^Psychiatric present status include hallucinatory paranoid state, psychomotor excitement, stupor, Residual schizophrenia, depressive state, manic state, delirium, twilight state and dementia symptom

^d^Specific therapies include housing services, tentative overnight stay at home before discharge, physical activities, training on the use of medications, group psycho-education, individual psycho-education, physical health management services, training on nutritional management, training on financial management, social skills training, cognitive behavioural therapy, wellness recovery action plan, cognitive remediation therapy, individual occupational therapy, family psycho-education, and services from peer-supporters

#### Patient-reported measures

The study assesses three patient-reported experience and outcome measures at all time points. The EuroQOL Five Dimensions and Five Levels (EQ-5D-5L) will be used as the primary measure of interest (primary exposure) corresponding to the primary aim of this study. The EQ-5D has been widely used in the area of mental health [42]. In addition, the scoring and language of the EQ-5D-5L have been validated in multiple countries, including Japan [43–45]. The Sheehan Disability Scale (SDS), which is a self-reported disability measure, is used to assess participants’ self-perceptions of problems in their lives. It consists of three items: work/academic activities, social activities, and family communication. Previous studies have confirmed that the Japanese version of the SDS has convergent validity with the Global Assessment of Functioning, good internal consistency, and extremely high test–retest reliability [46–48]. We will also employ a new scale to measure subjective views of empowerment and self-agency in people with mental illness. This scale asks participants whether they express themselves in their daily lives and whether they make decisions about the things that they encounter in their lives. The scale was tested in an assertive community treatment sample with relatively severe mental illness in Japan. Good factor validity has been confirmed [49]. Trained staff members help participants read and understand the sentences in each scale if necessary.

In addition to these repeated measures, the participants will complete an original questionnaire regarding their satisfaction with inpatient or outpatient care, subjective feelings about inpatient or outpatient care needs, and their relationships with family members at the time of discharge (T2) and 6-month follow-up (T3). Due to the paucity of brief, easily understandable, and scientifically validated scales designed specifically for assessing satisfaction with care and family relationships among people with mental illness in Japanese settings, we developed this original questionnaire through collaboration with service users who have experienced both inpatient and outpatient care (Additional file 1). We anticipate that participants will not be able to forthrightly answer these questions if staff members collect the questionnaire because the questions ask about the quality of care from staff members. Thus, the participants will complete the questionnaires without any help from staff members and mail them to the data center.

#### Clinical, social, and other exposure and outcome measures

At each assessment point, past hospitalizations (primary outcome), length of hospitalization, types of hospitalization (voluntary or involuntary), employment status, and social relationship status will be evaluated using medical records and participant interviews. In terms of other clinical variables, overall symptoms will be assessed based on previous studies conducted in the United States that evaluated the severity of symptoms using a 5-point grading scale [6, 7]. In addition, the level of functioning will be assessed using the Japanese version of the Personal and Social Performance scale (PSP-J) [50]. The PSP has been used by clinicians worldwide as a rating tool for social and general functioning [51, 52]. The presence or absence of the following problematic behaviors will be abstracted from medical records: physical violence, self-harm, suicidal behavior, self-neglect, substance abuse, water intoxication, nuisance behavior, and non-adherence to treatment. Staff members will also make clinical judgements regarding the risk of the emergence of each type of problematic behavior during the ensuing 6 months. In addition to the actual presence of such behaviors, risk judgements will be determined because they can influence treatment planning. Past studies have suggested that judgements related to the risk of violence, based on readily accessible clinical information such as age, sex, and recent history of violence and drug use, may have predictive accuracy comparable to judgments based on more detailed risk assessment tools [53, 54]. Before the study started, staff members received a brief training session on rating each past problematic behavior and risk estimate.

In addition, study staff will collect data on assessment point-specific variables. At the time of admission (T1), information on sociodemographic characteristics (sex, age, living condition, pension, types of medical and social service utilization), diagnoses, existence of a crisis plan, and past prescriptions will be gathered. We will also assess current psychiatric statuses that are likely to affect self-reported measures at admission (T1), including hallucinatory paranoid state, psychomotor excitement, stupor, residual schizophrenia, depressive state, manic state, delirium, twilight state, and dementia symptoms. In addition, the READMIT items (e.g., comorbidities, past emergency service use; see Vigod et al.) will be included as the participants’ demographic and background information [17]. At the time of discharge (T2), information on the patients’ prescriptions and types of care provided during hospitalization will be collected. During follow-up assessments (T3 and T4), the outpatient services and prescriptions that participants received during the previous 6 months will be recorded. All information will primarily be gathered through medical records. If the medical records do not include an assessment item, trained staff members will ask the participants to provide additional data. In addition, we will collect data on the number of social or welfare service agencies in the patient’s living area as an environmental geographical variable using the electronic Regional Mental Health Resources Analyzing Database (ReMHRAD, https://remhrad.ncnp.go.jp/), which can identify the number of service agencies in the patient’s local government area of residence.

#### Cost data

Supplemental cost data will be collected from two participating hospitals to illustrate costs during the study period, although we will not use these data in the main analysis. Medical costs will be gathered using individual receipt data from the two hospitals. To gather the cost data of medical services used in medical institutions other than the two hospitals and social services, we will assess the number of such services used through the Japanese version of the Client Service Receipt Inventory (CSRI-J) [55]. The CRSI-J includes the cost of social security and other public or social allowances in addition to information about services. At each assessment point, staff members at the two hospitals will interview the participants about their service utilization using CSRI-J. The costs of services will be computed in reference to a previous Japanese cost study [56]. Specifically, medical service and social service costs will be calculated based on the unit costs in the National Health Insurance or the Services and Supports for Persons with Disabilities Act, respectively. In addition, the costs per hour of services from public sectors (e.g., city offices, local government, and public unemployment offices) will be estimated based on the average salaries earned by public officers of the average age in Japan.

### Sample size consideration

Based on the primary aim of this study, the sample size was calculated using the effect size reported by a previous study. That study reported an association between readmission and QoL scores (*d* = 0.26) [20] and indicated the readmission rate in Japanese psychiatric hospitals (40%, ratio of approximately 1 vs 1.5) [38]. In addition, the calculation was based on a power of 70% at a two-sided significance level of 5%. Based on these values, 430 participants will be needed. Assuming that 20% of the participants will not be discharged within 12 months and a 25% attrition rate [57], the final number of participants required was estimated to be 624. This calculation was conducted using Stata version 15.

### Data analysis

#### Descriptive statistics

Descriptive summary statistics will be calculated for sociodemographic characteristics, outcomes, and other exposures. These values will be presented as means, standard deviations, medians, interquartile ranges, frequencies, and proportions, as appropriate.

#### Primary objectives

For the primary objective, mixed-effects logistic regression will be performed to assess the relationship between the QoL score at discharge (T2) and readmission over a 12-month follow-up period beginning at discharge after the index admission. In the first model, we will adjust for sex, age, and diagnosis. Next, we will conduct an analysis that adjusts for the potential covariates suggested in previous studies, for example, function (PSP score), symptoms, accommodation, problematic behaviors, past hospitalization, comorbidities, and utilization of outreach services after discharge, in addition to sex, age, and diagnosis. Statistical significance will be set at 5% (two-tailed). In these models, we will only include participants with data for both the primary exposure (QoL score at discharge, T2) and the primary outcome (readmission over 12 months).

#### Secondary objectives

We will again conduct mixed-effects logistic regression to assess whether self-reported disability, empowerment/self-agency-related measure or service satisfaction with inpatient care at discharge (T2) affect readmission over a 12-month follow-up period beginning at discharge after the index admission, respectively. In addition, we will conduct an analysis that adjusts for the same potential covariates as the QoL analyses. We will only analyze patients with data on self-reported disability (T2), empowerment/self-agency-related measure (T2) or service satisfaction (T2) with inpatient care and readmission.

#### Additional objectives

We will also create mixed models with repeated measures to explore the impacts of readmission on changes in QoL, empowerment/self-agency-related measure, and self-reported disability. In this model, we will adjust for sex, age, diagnosis, baseline score at admission (T1) and present psychiatric status at admission (T1). In addition, we will explore other potential factors that influence the changes in patient-reported outcomes using mixed models with repeated measures. In this analysis, we will include at least the following variables: function (PSP score), length of hospital stay, living condition, and utilization of outreach services after discharge. Because the mixed-model method can accommodate missing data, we will use the data from patient-reported measures completed at least twice at the three follow-up assessments (T2–T4), in addition to the assessment at admission (T1).

## Discussion

The present study will primarily examine the association between psychiatric readmission and patient-reported experiences, particularly QoL. Past studies have often focused on schizophrenia only or followed participants from the time of discharge. Apart from three German studies [11–16, 24, 30, 31, 58], few studies have followed participants who meet broad criteria from the time of admission to 12 months after discharge from the index admission to examine such an association. This study will provide new evidence regarding whether patients’ subjective feelings about their lives influence clinical outcomes. In addition, this study will collect various types of data regarding factors potentially associated with readmission and patient-reported measures at the individual and service levels. If patient-reported experiences are able to predict future readmission after adjustment for potential covariates, then routine clinical practice should focus more on each patient’s subjective perceptions of their illness and life. In addition, if improving patients’ subjective feelings or experiences can prevent readmission, it may also contribute to reducing the cost of inpatient care. Conversely, if we identify a particular factor that influences the course of patient-reported outcomes (e.g., QoL) in community living after discharge, it will provide useful evidence for improving clinical and social services in the community setting. In addition, we will suggest that mental health and governmental institutions routinely assess such variables and accumulate data. Such findings might contribute to a future large-scale study using existing data as the value of large-scale observational studies in health research has dramatically increased in parallel with the development of national databases [59].

We recognize some study limitations. The first is related to the nature of observational data. The research findings may suggest key factors affecting readmission and patient-reported outcomes at 12 months of follow-up, but the study will not be able to clarify the effect of a particular intervention. Second, the generalizability of study findings will be limited, even though the study will be conducted at multiple sites across Japan. Participating institutions are limited to hospitals that are relatively accessible for research work in a Japanese context. The third limitation is the sampling procedure that will be used for this study, particularly for those who have very severe psychiatric symptoms at the time of admission (T1). The participants will consist only of patients who can voluntarily consent to study participation and can complete the self-rated questionnaire. In other words, the study will not be able to include patients who cannot respond to the questionnaire, for example, due to very severe symptoms. Although we will adjust for diagnoses and symptoms that are likely to affect participants’ capacity to respond to patient-reported measures, the study will be affected by response bias. Measuring patients’ views of themselves, their lives, and their satisfaction with treatment may be challenging in the acute psychiatric care setting, but these evaluations are not impossible, as shown by a previous study [24]. We specifically selected brief, self-rated measures for this study to help address this problem. However, another potential limitation is the EQ-5D-5L, which is used as a patient-reported experience and outcome measure and is the primary interest in this study. While the scale is a validated measure, it is not a gold standard for assessing QoL, particularly in patients with schizophrenia or bipolar disorder [42]. However, the scale has several advantages, such as its brevity, simplicity, utilization of economic evaluation, and the finding that its results can be compared among people with other disorders or among the general population [60].

Despite the potential methodological limitations, this study attempts to collect data on multiple variables. It may be difficult to gather data for patient-reported experiences and outcome measures repeatedly in a retrospective or a national database study. In addition, our protocol was developed through collaboration among researchers, clinicians and service users. The findings of this study will be valuable for understanding patients’ views of hospitalization and community living.

### Dissemination of findings

The study findings will be reported based on the Strengthening the Reporting of Observational Studies in Epidemiology (STROBE) Statement [61] in peer-reviewed publications and presented at relevant scientific conferences. In addition, the results will be summarized and submitted to the funding body (Ministry of Health, Labour, and Welfare of Japan) to fulfil grant-reporting requirements. We will also ask an organization involving mental health service users (Community Mental Health & Welfare Bonding Organization, COMHBO) to help disseminate the study findings.

## Additional file

**Additional file 1.** Questionnaire for service satisfaction and subjective family relationship.

## Data Availability

The datasets generated and/or analyzed during the current study are not publicly available due to a relevant Japanese policy for Ethical Guidelines for Medicine for a person of interest and the ethical committee approval for this study but are available from the corresponding author on reasonable request.

## Abbreviations

CSRI-J: Japanese version of the Client Service Receipt Inventory
EQ-5D-5L: EuroQOL Five Dimensions and Five Levels
PSP: Personal and Social Performance scale
QoL: quality of life
SDS: Sheehan Disability scale
