# Supplementary material for: Associations between readmission and patient-reported measures in acute psychiatric inpatients: a study protocol for a multicenter prospective longitudinal study (the ePOP-J study)
Source: Int J Ment Health Syst. 2019 Jun 7;13:40. doi: 10.1186/s13033-019-0298-3 (PMC6555753; doi:10.1186/s13033-019-0298-3)
Supplement: Supplementary file 1 — Additional file 1. Questionnaire for service satisfaction and subjective family relationship. [file 13033_2019_298_MOESM1_ESM.docx]

**Questionnaire for service satisfaction and subjective family relationship**

**[Discharge]**

Please choose only **one option** for each question. Please put a checkmark in the box for your answer.

| Please rate your satisfaction with **this inpatient admission.** | | |
| --- | --- | --- |
| 1 | Would you recommend treatment at this hospital to people who are important to you if they need the same treatment you received? | □ I would recommend  □ I would probably recommend  □ I would probably not recommend  □ I would not recommend |
| Please answer the following questions about your overall satisfaction with **this inpatient admission.** | | |
| 2 | Are you satisfied with the attitude of your doctor?  (e.g. considerate, provided reasonable accommodation, and respectful) | □ I am satisfied  □ I am somewhat satisfied  □ I am somewhat not satisfied  □ I am not satisfied |
| 3 | Are you satisfied with the treatment by your doctor?  (e.g. medical examinations, prescriptions, etc.) | □ I am satisfied  □ I am somewhat satisfied  □ I am somewhat not satisfied  □ I am not satisfied |
| 4 | Are you satisfied with the attitude of staff members other than your doctor?  (e.g. considerate, provided reasonable accommodation, and respectful)  *staff = nurse, occupational therapist, psychiatric social worker, psychologist, etc. | □ I am satisfied  □ I am somewhat satisfied  □ I am somewhat not satisfied  □ I am not satisfied |
| 5 | Are you satisfied with the treatment by staff members other than the doctor?  (e.g. consultations, treatment programmes, etc.)  *staff = nurse, occupational therapist, psychiatric social worker, psychologist, etc. | □ I am satisfied  □ I am somewhat satisfied  □ I am somewhat not satisfied  □ I am not satisfied |
| 6 | Are you satisfied with the facilities and amenities?  (e.g. patient room, bed, toilet, bathing/showering tub, telephone, television, vending machine, notice board, etc.） | □ I am satisfied  □ I am somewhat satisfied  □ I am somewhat not satisfied  □ I am not satisfied |

| Do you agree with the following statements | | |
| --- | --- | --- |
| 7 | I needed this hospitalization | □ I agree  □ I somewhat agree  □ I somewhat do not agree  □ I do not agree |
| 8 | I need ongoing outpatient care after discharge | □ I agree  □ I somewhat agree  □ I somewhat not agree  □ I do not agree |

| Do you agree with the following statement | | |
| --- | --- | --- |
| 9 | Living with my family member(s) make me feel safe and comfortable | □ Yes, I agree  □ Yes, I somewhat agree  □ No, I somewhat do not agree  □ No, I do not agree |
| Please answer the following questions about your family members | | |
| 10 | Does your family member(s) understand your illness? | □ Yes, they do  □ Yes, they somewhat do  □ No, they somewhat do not  □ No, they do not |
| 11 | Does your family member(s) give you financial support? | □ Yes, they do  □ Yes, they somewhat do  □ No, they somewhat do not  □ No, they do not |
| 12 | Does your family member(s) keep a reasonable distance from you?  (They neither nag nor ignore you.) | □ Yes, they do  □ Yes, they somewhat do  □ No, they somewhat do not  □ No, they do not |

**Questionnaire for service satisfaction and subjective family relationship**

**[6 months after discharge from the index admission]**

Please choose only **one option** for each question. Please put a checkmark in the box for your answer.

| Please rate your satisfaction with the **outpatient care you are currently receiving.** | | |
| --- | --- | --- |
| 1 | Would you recommend the treatment from this hospital to people who are important to you if they need the same treatment you are receiving? | □ I would recommend  □ I would probably recommend  □ I would probably not recommend  □ I would not recommend |
| Please answer following questions about your overall satisfaction with the **outpatient care you are currently receiving.** | | |
| 2 | Are you satisfied with the attitude of your doctor?  (e.g. considerate, provided reasonable accommodation, and respectful) | □ I am satisfied  □ I am somewhat satisfied  □ I am somewhat not satisfied  □ I am not satisfied |
| 3 | Are you satisfied with the treatment by your doctor?  (e.g. medical examinations, prescriptions, etc.) | □ I am satisfied  □ I am somewhat satisfied  □ I am somewhat not satisfied  □ I am not satisfied |
| 4 | Are you satisfied with the amount of time you have to tell your doctor what you want to say during a medical consultation/examination? | □ I am satisfied  □ I am somewhat satisfied  □ I am somewhat not satisfied  □ I am not satisfied |
| 5 | Are you satisfied with the attitude of staff members other than the doctor?  (e.g. considerate, provided reasonable accommodation, and respectful)  *staff = reception staff, counselling room staff, nurse, occupational therapist, psychiatric social worker, psychologist, etc. | □ I am satisfied  □ I am somewhat satisfied  □ I am somewhat not satisfied  □ I am not satisfied |
| 6 | Are you satisfied with the environment of the waiting room and waiting times?  (e.g. crowdedness, notice board, sofa, toilet, lighting, music, vending machine, water cooler, etc.) | □ I am satisfied  □ I am somewhat satisfied  □ I am somewhat not satisfied  □ I am not satisfied |

| Do you agree with the following statements | | |
| --- | --- | --- |
| 7 | I needed the outpatient care I have been receiving for the past 6 months | □ I agree  □ I somewhat agree  □ I somewhat do not agree  □ I do not agree |
| 8 | I need ongoing outpatient care. | □ I agree  □ I somewhat agree  □ I somewhat not agree  □ I do not agree |

| Do you agree with the following statement | | |
| --- | --- | --- |
| 9 | Living with my family member(s) make me feel safe and comfortable | □ Yes, I agree  □ Yes, I somewhat agree  □ No, I somewhat do not agree  □ No, I do not agree |
| Please answer the following questions about your family members | | |
| 10 | Does your family member(s) understand your illness? | □ Yes, they do  □ Yes, they somewhat do  □ No, they somewhat do not  □ No, they do not |
| 11 | Does your family member(s) give you financial support? | □ Yes, they do  □ Yes, they somewhat do  □ No, they somewhat do not  □ No, they do not |
| 12 | Does your family member(s) keep a reasonable distance from you?  (They neither nag nor ignore you.) | □ Yes, they do  □ Yes, they somewhat do  □ No, they somewhat do not  □ No, they do not |
